# Costs of Introducing and Delivering HPV Vaccines in Low and Lower Middle Income Countries: Inputs for GAVI Policy on Introduction Grant Support to Countries

**DOI:** 10.1371/journal.pone.0101114

**Published:** 2014-06-26

**Authors:** Ann Levin, Susan A. Wang, Carol Levin, Vivien Tsu, Raymond Hutubessy

**Affiliations:** 1 Independent consultant to WHO, Washington D.C., United States of America; 2 Department of Immunization, Vaccines and Biologicals, World Health Organization, Geneva, Switzerland; 3 University of Washington, Seattle, Washington, United States of America; 4 PATH, Seattle, Washington, United States of America; University of California, San Francisco, United States of America

## Abstract

**Background:**

In November 2011, the GAVI Alliance made the decision to add HPV vaccine as one of the new vaccines for which countries eligible for its funding (less than $1520 per capita income) could apply to receive support for national HPV vaccination, provided they could demonstrate the ability to deliver HPV vaccines. This paper describes the data and analysis shared with GAVI policymakers for this decision regarding GAVI HPV vaccine support. The paper reviews why strategies and costs for HPV vaccine delivery are different from other vaccines and what is known about the cost components from available data that originated primarily from HPV vaccine delivery costing studies in low and middle income-countries.

**Methods:**

Financial costs of HPV vaccine delivery were compared across three sources of data: 1) vaccine delivery costing of pilot projects in five low and lower-middle income countries; 2) cost estimates of national HPV vaccination in two low income countries; and 3) actual expenditure data from national HPV vaccine introduction in a low income country. Both costs of resources required to introduce the vaccine (or initial one-time investment, such as cold chain equipment purchases) and recurrent (ongoing costs that repeat every year) costs, such as transport and health personnel time, were analyzed. The cost per dose, cost per fully immunized girl (FIG) and cost per eligible girl were compared across studies.

**Results:**

Costs varied among pilot projects and estimates of national programs due to differences in scale and service delivery strategy. The average introduction costs per fully immunized girl ranged from $1.49 to $18.94 while recurrent costs per girl ranged from $1.00 to $15.69, with both types of costs varying by delivery strategy and country. Evaluating delivery costs along programme characteristics as well as country characteristics (population density, income/cost level, existing service delivery infrastructure) are likely the most informative and useful for anticipating costs for HPV vaccine delivery.

**Conclusions:**

This paper demonstrates the importance of country level cost data to inform global donor policies for vaccine introduction support. Such data are also valuable for informing national decisions on HPV vaccine introduction.

## Introduction

### Background

Since 2009, the World Health Organization (WHO) has recommended that routine HPV vaccination for 9–13 year old girls be included in national immunization programmes in countries where: 1) the prevention of cervical cancer and/or other HPV-related diseases is a public health priority, 2) vaccine introduction is programmatically feasible, 3) sustainable financing can be secured, and 4) the cost-effectiveness of vaccination strategies in the country or region has been duly considered [1]. The recommended target population for HPV vaccine is 9 to 13 year old girls, a population that has not been routinely served by immunization programmes in most low or low middle income countries (LMICs). Thus, a decision to introduce HPV vaccine in such countries requires creation of new vaccine delivery services in order to deliver 3 doses to each girl. Unlike new infant vaccines which may be added to an existing infant vaccine delivery system, 9–13 year old children in many parts of the world currently receive limited or no routine preventive or other health services, so there is also limited or no existing preventive health service delivery system in place on which HPV vaccine delivery can depend. However, in some LMICs, HPV vaccination will be easier to introduce since school health programs are already in place in many countries and are already giving booster vaccinations. Thus, before introducing HPV vaccine, policymakers and program managers must understand the costs both of procuring the vaccine and of delivering the vaccine.

In November 2011, the GAVI Alliance made a decision to add HPV vaccine as one of the new vaccines for which countries eligible for its funding (less than $1520 per capita income) could apply to get national vaccination support, provided the applicant country could demonstrate the ability to deliver HPV vaccines [2]. GAVI’s new vaccine introduction support consists of providing a country with a supply of substantially subsidized new vaccine and with a one-time new vaccine introduction grant to contribute towards a country’s initial expenses of vaccine introduction. Until 2012, GAVI provided new vaccine introduction grants of $0.30 per infant in the surviving birth cohort of a country for any new vaccine, regardless of whether the vaccine was a substitution for a vaccine previously in the immunization schedule (e.g., combination Hib-DTP-HepB vaccine replacing DTP), new to the schedule and injectable (e.g., pneumococcal), new to the schedule and oral (e.g., rotavirus vaccines), and regardless of how many new vaccine doses were being added to the schedule. It has not been GAVI’s intent that the introduction grant covers the full costs of new vaccine introduction but “to facilitate the timely and effective implementation of critical activities in the national vaccine introduction plan in advance of a new vaccine introduction. and cover a share of the pre-introduction activities” [3].

The external HPV vaccine technical advisory group that developed the background technical briefs and advice to the GAVI Board for its HPV vaccine decision recognized that particular features of HPV vaccine delivery would make the vaccine more expensive to introduce and implement than a new infant vaccine. The technical advisory group therefore undertook a specific review and analysis of costs of HPV vaccine introduction and delivery. This paper describes the costing information available for the 2011 GAVI Board decisions related to 1) GAVI support for HPV vaccine introduction and 2) amount of the GAVI HPV vaccine introduction grant reviews. The paper includes review of how the delivery strategies and costs for HPV vaccine are different from other vaccines and what was known about the cost components from the data available in 2011 which originated primarily from HPV vaccine costing studies in low and middle income-countries.

It should be recognized that when the cost analyses were done in 2011, limited HPV vaccine delivery experiences for developing countries were available and no published literature existed on detailed program costs for HPV vaccines in these settings. In addition, there was no empirical data on the costs of introducing and scaling up HPV vaccination programs. The few studies that were underway at the time to estimate costs for HPV vaccine delivery in LMICs were part of various projects undertaken by different groups such as PATH [4], WHO [5], and the London School of Hygiene and Tropical Medicine (LSHTM) [6]; data from those works-in-progress were available and examined.

### Programmatic and cost considerations related to vaccine delivery

Optimal vaccine delivery strategies to routinely reach girls with multiple HPV vaccine doses in ways that are acceptable, affordable, and sustainable and which achieve high coverage are still being determined. Analyzing and understanding programmatic and cost components of vaccine delivery strategies currently used by immunization programmes for other vaccines can help provide insights useful for planning and addressing cost and sustainability issues related to HPV vaccine delivery.

Vaccination of infants generally occurs through “routine” vaccination services which typically consists of making vaccine available periodically (daily, weekly, or monthly) through consistent delivery services which may be a combination of fixed (e.g., health center) and outreach services, plus mobile services in some places. [7] From a programme planning perspective, these aspects of regular, periodic delivery services allow for consistent budgeting, consistent allocation of human and other resources, and establishment of persistent supportive infrastructure. From a patient or community perspective, the known regular and periodic availability of vaccine can allow for several opportunities for patient access and for vaccination on patient or parent demand. By contrast, vaccination that occurs through campaigns or Supplemental Immunization Activities (SIAs) generally is variably scheduled according to epidemiological, disease, and programme needs during a concentrated number of days to reach a target population with a same fixed vaccine or set of vaccines and other health services. Services are expanded for a temporary period to include not just fixed and outreach, but additional vaccination sites and sometimes door-to-door services. Campaigns may be nationwide or, as in the case of maternal-neonatal tetanus elimination, may target areas that are insufficiently covered by routine services. Campaigns require “surge capacity” in terms of human and financial resources to deliver vaccine. This need for surge capacity and the aspects of irregular or non-periodic vaccine delivery result in greater challenges for consistent budgeting, allocation of human and other resources (e.g., transportation, cold chain), and establishment of persistent supportive infrastructure. Additionally, vaccine delivery through campaigns typically provides intensive availability of vaccine but limited times to access the vaccine on patient or parent demand; some vaccines may have limited or no availability to the community outside of the time period of campaign delivery. HPV vaccine delivery strategies commonly employed in many low and low-middle-income countries with HPV vaccine activities currently have fewer characteristics of traditional “routine” vaccination since these are provided to a target population comprised of adolescent girls and are typically conducted as 3 scheduled brief vaccine delivery episodes that are more characteristic of campaigns from the programme and patient perspectives.

### Comparison of introduction and recurrent costs of delivering pneumococcal, yellow fever, and HPV vaccines

Table 1 qualitatively compares the introduction and recurrent costs of introducing HPV vaccine based on previous studies using 3 different primary delivery strategies with those of introducing other vaccines: 1) the three-dose pneumococcal vaccine (PCV) given to infants on the same schedule as other infant vaccines in health centres, and 2) the one-dose yellow fever vaccine delivered through a campaign. PCV vaccine costs are shown as the baseline costs and are assumed to have the least costly service delivery strategy. The three different HPV vaccine delivery strategies shown in the table are school-based periodic intensification of routine immunization (PIRI), outreach vaccine delivery PIRI integrated with other preventive health, and routine provision of health facility (fixed site)-based activities. The introduction costs for HPV vaccine compared to PCV were found to be [8] 1) higher for micro-planning (defined as planning of vaccination activities at local levels that take into account issues of accessibility, geography, population movements, and cultural characteristics) since these are targeted to an older age group, 2) about the same for training of vaccinators with the exception of school-based delivery, 3) higher for social mobilization/information, education and communications (IEC) due to the need to communicate about the different target population (different age group, specific sex) and delivery strategy as well as more extensive needs to provide education about the disease and the vaccine for HPV vaccine than for pneumococcal vaccine, 4) higher cold chain equipment requirements for delivery strategies outside of the health facility if there is a need to purchase additional equipment to transport vaccines; and 5) higher service delivery costs related to personnel time and allowances, supervision and monitoring and supervision for reaching the target population at schools or outreach posts.

**Table 1:** Qualitative comparison of Introduction and Recurrent Costs of PCV, Yellow Fever Vaccine, and HPV Vaccine.

	Pneumococcal Vaccine(routine, healthfacility)	Yellow FeverVaccine(campaign)	HPV Vaccine(periodic school-based)	HPV Vaccine(periodic integratedcampaign)	HPV Vaccine(routine healthfacility)
**Introduction Costs**					
**Micro-planning**	Baseline	+	++	++	++
**Training**	Baseline	=	+	=	=
**IEC and Social Mobilization**	Baseline	+	++	++	++
**Cold Chain Improvements**	Baseline	+	+	+	=
**Recurrent Costs**					
**Routine IEC and social mobilization**	Baseline	+	+	+	+
**Refresher Training**	Baseline	=	=	=	=
**Service Delivery** **(personnel time,** **outreach per diems,** **transport)**	Baseline	++	+++	++	+
**Monitoring and Evaluation**	Baseline	=	++	+	+
**Supervision**	Baseline	++	+++	+	+
**Waste Management**	Baseline	=	=	=	=

Note:  =  means equal, + means small increase in costs, ++ means medium increase in costs, and +++ means large increase in costs.

Yellow fever vaccination through campaigns is expected to be costlier than PCV vaccination since campaigns require more planning, social mobilization and cold chain equipment than a vaccine given at fixed sites through routine immunization at weekly or monthly vaccination sessions. Past studies [9] found that yellow fever campaigns were more costly than adding yellow fever vaccine to an existing immunization program. HPV vaccination is assumed to require more micro-planning, training where school-based delivery strategies are employed, and social mobilization and IEC than yellow fever vaccination since it is a newer vaccine, targeted only towards girls, and requires three doses.

## Methods

### Assumptions and data collection

To estimate the eligible population of girls for HPV vaccination, the authors took the population for the age chosen by the governments for HPV vaccine introduction – in most cases, girls ten years of age, except for the pilot study of girls vaccinated in the sixth year of primary school in Tanzania. For the pilot projects, the numbers were taken from administrative data on number of girls enrolled in school or number of girls in the target age group. For the scaled-up estimates, data from government population projections on the number of girls in the designated age group were used. The data on number of girls for the grade based school vaccination were taken from school enrollment lists.

In order to evaluate the cost structure of HPV vaccine service delivery, financial costs from various studies were compared. Financial costs are costs to the payer (i.e. Ministry of Health) of HPV vaccine introduction and service delivery and include the value of actual resources purchased. For example, some financial cost categories include injection supplies, outreach allowances, and resources used in training and development of new communication materials, supervision, and monitoring and evaluation. Financial costs are a sub-set of economic costs since the latter also include resources used for service delivery that have opportunity costs – i.e., those already paid for or owned by the Ministry of Health, such as salaries of health personnel, partner-donated items such as vaccines, and volunteer time. A summary of the main assumptions made in the analysis are shown in Table 2.

**Table 2:** Assumptions made in HPV Vaccination Cost Analysis.

Variable	Assumption
**Target Population**	Defined either one single year of age cohort of girls between nine and thirteen years old or one single school grade cohort of girls
**Number of doses**	3 dose schedule administered during the course of 6 months
**Dropout rates**	Percent of target population that gets the first dose but not the second dose or percent of target population that get the second dose but not the third dose*
**Coverage**	Number of girls fully vaccinated divided by the total girls in the designated age group or grade
**Introduction Costs**	Micro-planning, IEC/social mobilization, training, and purchase of cold chain storage/equipment.
**Recurrent Costs**	Routine social mobilization and IEC, refresher training, service delivery (outreach per diems, transport), monitoring and evaluation, supervision, and waste management

*Definition of dropout rates is from Bos (2000). Using Immunization Coverage Rates for Monitoring Health Sector Performance: Measurement and Interpretation Issues”, August 2000 HNP Discussion Paper. World Bank.

Two categories of costs were estimated for this analysis: introduction or initial investment costs (sometimes also referred to as start-up costs) and recurrent (also referred to as operational) costs. Introduction costs are treated separately from recurrent costs since these are capital costs and can be used over a period greater than one year. The introduction costs are for those investments that occur during the initial years of the introduction (some may occur during the second or third year if the introduction is phased in the country) and typically include investments in additional cold chain if needed, planning activities for a new vaccine, sensitization of national and sub-national health officials, community sensitization, development of new communication and training materials and guidelines, and one-time training on the new vaccine. To get average introduction costs, the total costs were divided by the number of eligible girls (girls in the target population) rather than by girls actually vaccinated since planning, training and social mobilization and IEC is conducted with the intent of reaching the entire target population.

Second, recurrent costs are the running costs of the program such as transport, allowances, monitoring and evaluation, and supervision. To get average cost, total introduction and recurrent costs are divided by number of vaccinations or girls that received three doses to get cost per dose, cost per fully immunized girl (FIG) and cost per eligible girl.

For this analysis, data on service delivery costs from pilot demonstration projects [4], [10], national scaling-up estimates, and national expenditure data were analyzed. In order for the data from these different sources to be comparable, all of the initial investment costs were assumed to occur in the first year even though vaccine introduction sometimes takes place over more than one year.

The costs of scaling up HPV vaccination nationwide were estimated for two African countries: Tanzania and Uganda. In Tanzania, the costs of nationwide introduction were estimated using the WHO Cervical Cancer Prevention and Control Costing (C4P) Tool [4]. Tanzania had planned to introduce HPV vaccine in 2013–14 and all the assumptions and data requirements for school-based and health facility based strategies were entered into the costing tool to get the cost estimates for each strategy. In Uganda, average costs from a demonstration project in two districts, Ibanda and Nakasongola in Western and Central Uganda, respectively [3], were used to estimate nationwide financial resource requirements for HPV vaccination introduction through participation in periodic activities known as Child Health Days.

### Data Collection

Three types of costs and expenditures were used for this analysis. First, cost data were assembled from pilot projects in five countries: India, Peru, Uganda, Vietnam, and Tanzania (see [4]–[6] for details). Second, costs were estimated for nationwide HPV vaccination in Tanzania with the C4P tool [5] and existing estimates were adapted for Uganda along with assumptions about resource use and costs of scaling-up. Third, country expenditure data were obtained in 2011 from the Bhutan national immunization program for its 2010 national HPV vaccine introduction [11].

## Results

The cost estimates are shown in Tables 3 and 4 for pilot projects and in Table 5 for nationwide scale-up. These estimates differ in types of costs included and the resource-intensity of implementation. That is, more health personnel time and effort (measured through per diem and transport allowances in financial costs) is often put into pilot projects than would occur on a nationwide level. Importantly, the cost estimates for pilot projects do not include some costs of a national program, such as monitoring and evaluation. These pilot projects also are often conducted in more accessible and better functioning areas and may under-represent the costs of more remote or low-performing areas. Thus, these may underestimate more typical introduction and recurrent (operational) costs.

**Table 3:** Financial costs for HPV vaccine introduction and delivery in pilot projects in selected countries.

Country	Tanzania(LSHTM pilot project)Age-basedarm	Tanzania(LSHTM pilot project)Grade-basedarm	Uganda(PATH HPVvaccinedemonstrationproject)	Uganda(PATH HPVvaccinedemonstrationproject)	India-AP(PATH HPVvaccinedemonstrationproject)	India-Gujarat(PATH HPVvaccinedemonstrationproject)	Peru(not GAVI eligible)(PATH HPVvaccinedemonstrationproject)	Vietnam(PATH HPVvaccinedemonstrationproject)	Vietnam(PATH HPVvaccinedemonstrationproject)
	T1	T2	U1	U2	I1	I2	P1	V1	V2
**Currency**	US$ 2011	US$ 2011	US$ 2009	US$ 2009	US$ 2009	US$ 2009	US$ 2009	US$ 2009	US$ 2009
**Age of target** **population**	Girls born in 1998	Primary 6	10 yrs	P5/10 yrs	10 yrs	10 yrs	10 yrs	10 yrs	10 yrs
**Number of girls in** **target population**	2,180	3,352	2,263	3,459	14,533	12,636	8,092	1,890	1,205
**Delivery strategy**	Periodic School-based	Periodic School-based	Outreach	Periodic School-based	Periodic Schoolbasedin three rounds andoutreach AngawadiCenter (AWC)	Monthly in schools &Angawadi Center(implemented as periodicin practice)	Periodic School based	Periodic School based	Health center based
**Vaccine coverage** * **(1^st^ dose/3^rd^ dose)**	82%/72%	86%/79%	73.4%/52.6%	96.8%/90.5%	Urban 79.2%/77.2%Rural 89.6%/87.8%Tribal 86.2%/83.9%	Urban 70.1%/68.4%Rural 84.2%/83.3%Tribal 81.7%/74.4%	83.9%/82.6%	96.8%/96.1%	99.1%/98.6%
**Introduction costs** **per eligible girl** **	$6.09	$6.09	$6.61	$6.70	$1.49	$2.19	$11.45	$18.94	$18.44
**Recurrent costs** **per dose**	$5.64	$3.69	$0.56	$1.24	$0.32	$0.36	$0.63	$0.46	$0.42
**Recurrent costs** **per fully immunized** **girl** ***	$13.08	$9.18	$3.45	$3.97	$1.00	$1.05	$1.90	$1.42	$1.46

*Vaccine coverage across projects are not directly comparable since denominators for Tanzania projects only included school-enrolled girls and not all girls living in a district. Others are based on population-based coverage surveys.

**Introduction/initial investment costs include training, IEC/social mobilization, and other capital costs such as additional cold chain storage.

***Recurrent costs per fully immunized girl  =  [cost per dose×(total # of doses 1+doses 2+doses 3 delivered)]/number of girls who received three doses (as a function of coverage and dropout from one to three doses).

**Table 4:** Costs of Pilot Projects by Country Characteristics.

Countrycharacteristics	Country	IntroductionCosts	Recurrent costs perfully immunizedgirl	Comments
**Large country with high population density**	India AP; periodic school basedand periodic health center-based(PATH project)	$1.49	$1.00	Costs were likely lower due toeconomies of scaleand integrated services
	India Gujarat; Routine –periodic school-based andperiodic health center(PATH project)	$2.19	$1.05	
**Large country with high** **population density and** **more resource intensive** **immunization infrastructure**	Vietnam; Periodicschool based(PATH project)	$18.94	$1.42	Start-up costs were likelyhigher due to resource intensity andtraining and social mobilizationactivities are not integrated
	Vietnam; RoutineHealth center based(PATH project)	$18.44	$1.46	
**Less densely populated countries**	Tanzania; Periodic School-based (LSHTM project)	$9.69	$15.69	Costs were likelyhigher due to fewereconomies of scale
	Uganda; PeriodicIntegrated Outreach(PATH project)	$6.61	$3.45	
	Uganda; PeriodicSchool based(PATH project)	$6.70	$3.97	
**Middle-income country with** **better infrastructure**	Peru; PeriodicSchool based(PATH project)	$11.45	$1.90	Costs of start-up were likelyhigh due to intensive training andsocial mobilization.

*Integrated with Child Days Plus for doses 1 and 3.

**Table 5:** Financial costs of scaling-up HPV vaccine delivery in Tanzania, Uganda and Bhutan.

	Tanzania (C4P costing tool*) Program scaled-up	Tanzania (C4P costing tool*) Program scaled-up	Uganda (financial assessment to scale up from bridging study)	Bhutan national introduction with catch-up (based on expenditure data and 90% coverage)***	Average costs from prior four columns (not weighted by population)
**Type of Estimation**	Projected	Projected	Projected	Actual	
**Currency**	US$ 2011	US$ 2011	US$ 2011	US$ 2010	
**Age of target population**	10 yrs	10 yrs	10 yrs	12–18 yrs	
**Number of girls in target population**	605,000	605,000	675,270	47,888	
**Delivery strategy**	Periodicschool-based	Periodic healthfacility based	Periodic Integratedoutreach	Periodic school-based	
**Introduction costs per eligible girl** ****	$3.07	$3.07	$2.82**	$3.02	$2.99
**Recurrent costs per dose**	$1.59	$1.17	$1.27	$1.50	$1.38
**Recurrent costs for three doses per eligible girl**	$4.78	$3.51	$3.81	$4.56	$4.17

*The C4P tool is the WHO Cervical Cancer Prevention and Control Costing Tool.

**Uganda’s estimate is the introduction cost per girl for scaling up to all districts, following the demonstration project–where there was already an investment in start-up activities.

***Estimates for Bhutan were obtained from available country expenditure data from the national introduction with catch-up vaccination rather than from a full costing through the collection of resource and cost data. Costs would probably vary upon switching to a national vaccination of a single cohort per year through facility-based services.

****The calculation of cost per eligible girl divides the costs of the program with full coverage (100%) by the total number of girls in the target population.

### HPV Vaccination Pilot Projects

Table 3 shows the financial costs of these pilot projects by country and strategy. The average introduction costs per fully immunized girl range from $1.49 in India for outreach to $18.94 in Vietnam for a periodic school-based delivery strategy. The recurrent costs per fully immunized girl range from $1.00 in India for an outreach delivery strategy to $13.08 for a school-based approach in Tanzania.

In two countries, Uganda and Vietnam, the pilots had two arms where a periodic school-based strategy and an outreach or health facility strategy were compared. The cost of implementing the school-based delivery was greater for recurrent costs in Uganda and for introduction costs in Vietnam.

The variation in financial costs of pilot projects can be partly explained by country characteristics, such as population density and proximity of health facilities to schools, as well as the project design, extent of integration of services in existing programs and the level of intensity of effort. Table 4 shows some of the characteristics of countries that likely affect the costs of their pilot vaccination projects: size of country, population density, current infrastructure of schools and health facilities, and national income level. For example, introduction costs were highest in Vietnam and lowest in India. In the former, standalone training and planning occurred and in the latter, training and planning were integrated into existing immunization program activities. The recurrent cost per dose was highest in Tanzania and lowest in India. Recurrent costs were higher in the Tanzanian study arms since the research study project introduced higher transport and storage costs than may have been observed if integrating service delivery using government systems. If the scaled-up estimates are used (see [6]), these are more similar to those of the other pilot projects.

The two Asian countries, India and Vietnam, have relatively low recurrent costs which may be due to having large populations with high population density, which allow close proximity between health facilities and vaccination sites and efficiency and synergistic gains could be expected. The introduction costs were different for the two countries since India’s services were integrated with other health activities while Vietnam’s services were not. The Vietnamese government had a more resource intensive approach to introducing the vaccine for facility and school based strategies – i.e. specifically for micro-planning and training [4], [12].

The two African countries, Tanzania and Uganda, were more sparsely populated, with greater distances between health centers and vaccination sites. Recurrent costs were higher since costs of transport and/or allowances are greater. On the other hand, Peru, a middle-income country with better infrastructure, had greater introduction costs and higher salaries, intensive social mobilization and training costs.

### Estimates of Nationwide Scale-up

National scale-up cost and expenditure estimates are shown for three countries – Tanzania, Uganda and Bhutan - in Table 5. The total introduction costs per eligible girl range from $2.82 for a hybrid Child Health Day and periodic school-based strategy to $3.07 for either a periodic school- or routinely available health facility based strategy in Tanzania. These costs did not vary widely among the three countries.

### Comparisons between pilot and nationwide scale-up

Total introduction costs per eligible girl were higher for pilot projects than nationwide introduction, except for the India projects where services were integrated. However, total costs per eligible girl and per fully immunized girl were lower than nationwide introduction since the latter included more cost elements (such as national micro-planning, development of training and IEC materials, and training of trainers) but was spread over a larger population and was less resource-intensive.

Average recurrent financial costs for nationwide introduction ranged from $3.51 per eligible girl for implementation of HPV vaccination through health facilities in Tanzania to $4.78 for HPV vaccination through schools in Tanzania. Similar to introduction costs, the recurrent costs for nationwide scale-up differed from those of pilot projects, since more national costs such as supervision and monitoring and evaluation are included than would be found in pilot projects. In addition, two out of three of these countries are African and have lower population density, driving up the recurrent costs.

In Tanzania, the costs of two service delivery strategies were compared. The projected recurrent costs for periodic school-based administration were estimated to be greater than routinely delivered health facility-based vaccination due to the additional transport and outreach allowances associated with the former strategy.

## Discussion

This overview of costs for HPV vaccination found considerable variation in costs among pilot projects due to differences in 1) scope and scale (number of girls in target population, population density); 2) strategy (outreach, school-based, health facility); 3) national income levels and related public health cost, infrastructure and salary structures; and 4) health system policies and program – e.g. level of health service integration. Less variation is found for costs of nationwide scaling-up, as can be seen in Table 6. Based on this review of data available in 2011 for nationwide scaling-up for HPV vaccine, the average cost for introduction per eligible girl was $2.99 (range, $2.82–$3.07) and recurrent cost to deliver 3 doses per eligible girl was $4.17 (range, $3.51–$4.78). Nationwide scaling-up programs are spread over larger populations and are less intensive than pilot projects.

**Table 6:** Ranges of Introduction and Recurrent costs for different scenarios.

	Pilot Project(n = 7)	Pilot Project(n = 2)	Scaling-upHPV vaccine(n = 3)	Scaling-upHPV vaccine(n = 1)
**Source**	LSHTM 2 arms, PATH Uganda,India 2 arms, Peru, Vietnam	Uganda Child DaysPlus, Vietnam	Tanzania C4P, Uganda,Bhutan	Tanzania C4P
**Delivery Strategy**	School based periodic ordelivered monthly (India)	Health center/periodic	School based	Health Facility
**Range of introduction** **costs per** fully immunized girl*	$1.49–$18.94	$6.61–$18.44	$3.13–$5.15	$5.15
**Range of recurrent costs** **per dose**	$0.32–$5.64	$0.42–$0.56	$1.27–$1.67	$1.12
**Range of recurrent costs** **per fully** **immunized girl**	$1.00–$13.08	$1.46–$3.45	$4.23–$5.81	$5.27

Note: Recurrent costs per fully immunized girl  =  [cost per dose×(total # of doses 1+doses 2+doses 3 delivered)]/number of girls who received three doses (as a function of coverage and dropout from one to three doses).

Results from both pilot projects and nationwide scaling up indicate that periodic school-based strategies are costlier than integrated outreach or health center-delivery of HPV vaccine, since more introduction and recurrent costs are required for the former. Evaluating delivery costs along the programme characteristics (more routine-like versus more campaign-like) as well as by the country characteristics (population density, income/cost level, existing service delivery infrastructure) and the vaccine requirements (number of doses per schedule, injection vs oral, etc.) are likely the most informative and useful for anticipating costs for vaccine delivery.

The choice of service delivery strategies that is affordable will differ depending on the local context such as school enrollment levels and differences in geographical terrain as well as the availability of vaccine delivery infrastructure. If school attendance rates are low and non-school delivery strategies are particularly necessary, then an outreach strategy may be the best approach. In countries with low population density (e.g. small islands such as the Seychelles or mountainous areas of Nepal and Bhutan), the government may consider a strategy using mass vaccination strategies once every 3–5 years.

These data provided useful benchmarks for the range of possible costs of vaccine delivery for HPV vaccine introduction in terms of start-up and ongoing operational or recurrent costs and informed GAVI’s decision to set its HPV vaccine introduction grant to countries at $2.40 per eligible girl in the target population. GAVI introduction grants to countries are not intended to fully cover the introduction costs, but simply to contribute to addressing the costs of the first year.

The study had some limitations. It was sometimes difficult to compare data due to variation in study methods. The data generated by the WHO C4P Tool for Tanzania was based on assumptions and was prospective since the HPV vaccine had not yet been introduced into the country. However, the findings on cost per fully immunized girl were independently similar to the findings generated using actual pilot project data in Uganda to estimate national HPV vaccine delivery costs for Uganda.

The challenge for low and middle income countries is to secure financial resources to cover the delivery cost of HPV vaccines. A better understanding of the amount needed to scale up costs for high coverage of girls nationwide, including those who might be difficult to reach, and the cost structure of what drives these nationwide delivery costs is crucial for better planning for sustainable HPV vaccine introduction in LMICs.

This paper demonstrates the importance of country level cost data to inform both national and global policies on HPV vaccine introduction. Collecting the results of economic evaluations and presenting it in a systematic and standardized fashion to influence policy decisions is a useful way to ensure that international health interventions are based on evidence ([13], [14], [15], [16]).
